# Vaccination against tuberculosis, polio and hepatitis B at birth in Podor health district, Northern Senegal: cross-sectional study of vaccination coverage and its associated factors

**DOI:** 10.1186/s12889-022-12535-z

**Published:** 2022-01-15

**Authors:** Oumar Bassoum, Ndeye Mareme Sougou, Mouhamadou Faly Ba, Malick Anne, Mamoudou Bocoum, Alioune Dieye, Cheikh Sokhna, Anta Tal-Dia

**Affiliations:** 1 Service de Médecine Préventive Et de Santé Publique, Faculté de Médecine, de Pharmacie Et d’Odontologie (FMPO), Université Cheikh Anta Diop (UCAD), Dakar, Sénégal; 2 Institut de Santé Et Développement, Université Cheikh Anta Diop, Dakar, Sénégal; 3 Ministère de La Santé Et de L’Action Sociale, Dakar, Sénégal; 4 Service d’Immunologie, FMPO, UCAD, Dakar, Sénégal; 5 Institut de Recherche Pour Le Développement, Campus UCAD/IRD de Hann, Dakar, Sénégal; 6 Institut Hospitalo-Universitaire - Méditerranée Infection, Marseille, France

**Keywords:** Vaccination Coverage, Timely, Birth Doses, Senegal

## Abstract

**Background:**

In Senegal, studies focusing specifically on vaccination coverage with the Bacille de Calmette et Guérin (BCG) vaccine, the birth dose of oral polio vaccine (OPV zero dose) and the birth dose of hepatitis B (HepB-BD) vaccine are insufficient. This study aimed to highlight vaccination coverages with birth doses and factors associated with timely vaccination in Podor health district.

**Methods:**

A cross-sectional study was carried out from June 19 to 22, 2020. The study population consisted of children aged 12 to 23 months of which 832 were included. A stratified two-stage cluster survey was carried out. The sources of data were home-based records (HBR), health facility registries (HFR) and parental recalls. Timely vaccination refers to any vaccination that has taken place within 24 h after birth. Descriptive analyzes, the chi-square test and logistic regression were performed.

**Results:**

The crude vaccination coverages with BCG, OPV zero dose and HepB-BD were 95.2%, 88.3% and 88.1%, respectively. Vaccination coverages within 24 h after birth were estimated at 13.9%, 30% and 42.1%, respectively. The factors associated with timely HepB-BD are delivery in a health facility (AOR = 1.55; 95% CI = 1.02–2.40), access to television (AOR = 1.63; 95% CI = 1.16–2.29), weighing (AOR = 3.92; 95% CI = 1.97–8.53) and hospitalization of the newborn immediately after birth (AOR = 0.42; 95% CI = 0.28–0.62).

**Conclusion:**

Timely administration of birth doses is a challenge in the Podor health district. The solutions would be improving geographic access to health facilities, involving community health workers, raising awareness and integrating health services.

**Supplementary Information:**

The online version contains supplementary material available at 10.1186/s12889-022-12535-z.

## Introduction

Vaccination is a preventive act whose objective is to allow the vaccinated individual to benefit from specific protection against an infectious agent before any exposure to it [1]. It is recognized that apart from clean water and sanitation, vaccination has made the greatest contribution to global health, especially in developing countries [2]. Its principle is based on the induction of long-lasting and effective protection against a pathogen responsible for an infectious disease without causing clinical symptoms or side effects [3].

In 1974, the World Health Organization (WHO) established the Expanded Programme on Immunization (EPI). WHO recommends that the Bacille de Calmette et Guérin vaccine, the birth dose of oral polio vaccine (OPV-zero dose) and the birth dose of hepatitis B (HepB-BD) vaccine be given at birth, ideally within 24 h after birth [4–6]. These recommendations are motivated by the heavy burden of tuberculosis, hepatitis B and polio. According to the Global tuberculosis report, published in October 2020, around 10 million people contracted tuberculosis during the year 2019. The number of deaths was 1.4 million [7]. The African Region is one of the most affected regions and is home to 25% of new cases [7].

According to the Global Hepatitis Report published in 2017, 257 million people were living with chronic hepatitis B in 2015, a prevalence of 3.5% [8]. A recent mathematical modeling, published in 2018, showed that the number of people with hepatitis B was 291,992,000 in 2016; which corresponds to a prevalence of 3.9% [9]. Much of the disease-related morbidity results from transmission during childbirth or during infancy. Progression to chronic hepatitis is more common when infection occurs in these circumstances [6].

According to WHO, 10 to 20 million people of all ages are living with paralytic polio [10]. However, five of WHO's six regions, including Africa, are declared free of wild poliovirus and global polio eradication is approaching [11]. To date, transmission of wild poliovirus continues in only two countries, Pakistan and Afghanistan [12].

By 2030, the WHO estimates that vaccine coverage with BCG and HepB-BD followed by subsequent doses of hepatitis B vaccine reaching 90% should prevent 84% of hepatitis B virus-related deaths [6] and 115,000 tuberculosis-related deaths per birth cohort during the first 15 years of life [4]. Administration of OPV-zero at birth strongly contributes to the improvement of seroconversion rates after administration of subsequent doses [5, 13].

A Systematic Review and Meta-Analysis showed that in sub-Saharan Africa the pooled coverage rates at day 0–1 after birth were 14.2% (95% CI: 10.1–18.9) for BCG and 1.3% (0.0–4.5) for HepB-BD. No data were available for OPV0 at day 0–1. The rates of vaccine coverage immediately after birth were very low for BCG and HepB-BD, and no data for OPV0 [14]. This can be explained by several factors. Of these, human resource factors e.g., staff shortages, lack of training opportunities, poor attitude and gaps in knowledge among healthcare staff are frequently associated with poor uptake of immunization programs in Africa [15]. For population factors, effective vaccination outcomes are not only dependent on accessibility, which remains a major challenge in Africa, but also on the acceptance and willingness of the population to be vaccinated [16]. Thus, one of the major obstacles to achieving high immunisation coverage is vaccine hesitancy [17], which is defined by the World Health Organization (WHO) as delayed acceptance or refusal of vaccination despite the availability of immunisation services [18].

In Senegal, the tuberculosis incidence rate is estimated at 117 cases per 100,000 inhabitants in 2019. Children aged 0 to 14 years old accounted for 4% of the tuberculosis cases detected in the country [19].

Regarding hepatitis B, Senegal belongs to the group of countries with high endemicity [20]. It has been established that 85% of the general population have at least one HBV marker [21]. The prevalence in the Senegalese general population is estimated at 11% [22].

Senegal has implemented the EPI since 1979. In accordance with WHO recommendations, the country has a vaccination policy against tuberculosis, hepatitis B and polio at birth. The HepB-BD has been integrated into the EPI since February 2016 [23]. However, administration of this dose at birth faces many constraints such as home births and cold chain failures and, in some sub-Saharan Africa countries, lack of funding [24].

According to the continuous demographic and health survey carried out in Senegal in 2019 (CDHS-2019), vaccination coverage with BCG, OPV-zero dose and HepB-BD were respectively 94.5%, 80.5% and 81.3%. In the northern zone to which Podor health district belongs, these indicators are respectively estimated at 93.9%, 75.3% and 75.5% [25]. Administrative data in Podor health district indicate vaccination coverage with BCG and HepB-BD reaching respectively 98% and 51% in 2019 [26]. However, these data have shortcomings. The first concerns the CDHS-2019, the results of which do not clearly indicate vaccination coverage within 24 h after birth. The second shortcoming relates to administrative coverage data which estimate vaccination coverage with an imprecise denominator. This denominator corresponds to the number of children of eligible age, that is, an estimate of the target population for vaccination which can be obtained from a census [27]. Finally, both CDHS-2019 and administrative data did not assess factors that may influence vaccination coverage at birth.

It is in this context that this study was undertaken to help fill these gaps. The purpose is to allow the development of guidance notes in terms of strategies for improving vaccination coverage at birth. The objective of this study was to highlight vaccination coverages with birth doses and factors associated with timely vaccination in Podor health district.

### Conceptual framework

**Fig. 1:**
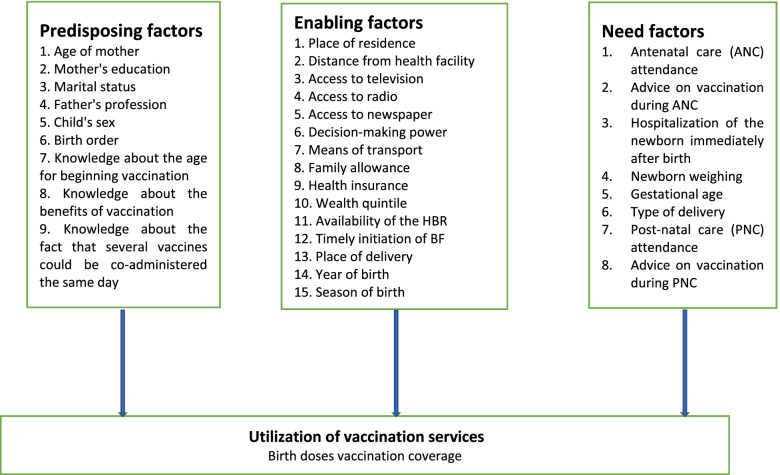
Conceptual framework

Vaccination at birth is linked to access and use of health services. Several studies use Andersen RM theoretical model to understand the factors that condition these events [28]. This present study fits into this logic in order to understand the factors that are associated with the timely administration of the birth dose vaccines. Thus, on the basis of a review of the literature, variables were identified and classified into three categories as recommended by this theoretical model (Fig. 1). Predisposing factors relate to socio-demographic and biological factors in Fig. 1. enabling factors refer to the financial and organizational resources available to individuals to access health services as shown in Fig. 1. Finally, the need factors are those that increase the use of services as shown in Fig. 1.

### Study setting

The study took place in Podor health district, which belongs to the eponymous department and Saint Louis region. The health district is located in northern Senegal, in the middle valley of the Senegal River and about 490 km from the Senegalese capital, Dakar. It is bounded to the north by the Islamic Republic of Mauritania, to the south by the health districts of Dahra and Linguère which appear to Louga region, to the west by the health district of Dagana and to the east by the health district of Pété. The health district hosts 247,891 inhabitants. The density is estimated at 35 inhabitants per km^2^ [29]. Much of the region's economic activity relies on agriculture and livestock [30].

It has been established that 45% of the population is in a mobile or advanced zone, that is to say more than 5 km from a health facility [29]. The district is home to a hospital, two health centers, one of which is non-functional, and thirty-eight health posts [29]. The health district is marked by a deficit in ambulances and motorcycles [29]. Immunization services are offered through three strategies. First, the fixed strategy is practiced at the level of health facilities and concerns the target population living within a radius of 5 km. Second, the advanced strategy is carried out at the level of health huts and sites by the staff of the health facilities. It is aimed at the population of localities located between 5 and 15 km from a health facility. Third, the mobile strategy targets the population living beyond 15 km from a health facility [23].

## Methodology

### Type, period and study population

It was a cross-sectional study. Data collection took place from June 19 to 22, 2020. The study population consists of children aged 12 to 23 months. This choice is based on the recommendations of the WHO, which considers that the assessment of routine immunization coverage should relate to an annual birth cohort. Children aged 12 to 23 months are the ideal population for estimating recommended dose coverage between 0 and 11 months. This period concerns not only the three birth doses but also other basic antigens, the last of which is given at 9 months, namely the measles-rubella and yellow fever vaccines. Another advantage is the possibility of estimating the full immunization coverage with this study population [31].

### Sampling

#### Eligibility criteria

The eligibility criteria are:

- Being 12 to 23 months old at the time of the survey;

- Having slept in the household the previous night.

#### Sample size

The sample size is estimated according to the new WHO recommendations [31].

First, the effective sample size (ESS) is calculated as follows:

$$n\ge \frac{k {z}_{1-\frac{\alpha }{2}}^{2}}{{4d}^{2}}+\frac{1}{d}-2 {z}_{1-\alpha /2}^{2}+\frac{{z}_{1-\alpha /2}+2}{k}$$

- Z_1-α_: standard normal distribution evaluated at 1-α = 5%

- d: desired half-width of the set confidence interval = 5%

- p: expected vaccination coverage rate = 90%

- k = 0.51. WHO estimates that when the expected immunization coverage rate is between 0.7 and 1-d, k is calculated using the following formula: k = 4 (p-d) (1-p + d) = 0.51.

Thus, the ESS is equal to 216 children aged 12 to 23 months. It corresponds to the number of subjects necessary for a simple random survey.

This size will be increased due to the variability between the clusters. To do this, it will be multiplied by a factor called the design effect (DEFF).

DEFF = 1 + (m—1) X ICC.

- m: number of subjects per cluster equal to 10.

- ICC: intra-cluster correlation coefficient fixed here at 1/3.

DEFF = 1 + (10—1) X 0.333 = 4.

Thus, the minimum sample size = ESS x DEFF = 864. This number is rounded to 870.

#### Number of clusters

The number of clusters to select is calculated by relating the sample size to the number of children required for each cluster.

$$\frac{870}{10}=87$$

Then, a distribution proportional to the size was made to determine the number of clusters required for each health structure.

#### Sampling procedure

A two-stage cluster sampling was carried out. The survey was stratified according the health facilities. This means that sampling was carried out in each stratum independently. All 39 health facilities were included and each corresponds to a geographical area covering several villages. The first stage corresponds to villages and / or neighborhoods (primary units), the number of which is allocated proportionately according to the size of the geographical area in terms of number of inhabitants. The selection was made according to a simple random survey using the ALEA function of the Excel software. The second stage is made up of households (secondary units). In each village or neighborhood included, households are visited. The first included household is randomly selected from the center of the cluster. Beforehand, a street near the centre of the cluster is drawn at random. The survey started with the first household on the right. The subsequent households are recruited step by step until the required number of children per cluster is reached.

A household may not be included due to unavailability or refusal to participate. In this case, it is replaced by the next household. In accordance with the principle of the cluster survey, all eligible children in a household are included.

### Data Collect

#### Survey tool

The survey tool was a structured questionnaire designed on the basis of the literature [31, 32]. Its validation followed several procedures. First, the research team reviewed and commented on it. Then, a pre-test was carried out in three districts of Podor. The collected Open Data Kit (ODK) application downloaded to smartphone tablets (Android) served as the data collection medium. The entered forms are sent to a server. An internet connection is required for downloading the application and submitting forms, but not for collecting in the field.

The pre-test is carried out on 80 children aged 12 to 23 months living in three parts of Podor city for purposes of inducing interviewers to become familiar with the survey tools. The comments resulting from the pre-test made it possible to finalize the questionnaire which has five sections: i) socio-demographic characteristics of the parents, ii) individual characteristics of the child, obstetric characteristics, iii) knowledge about vaccination, iv) vaccination data according to the home-based records (HBR) or the health facility registry (HFR) and v) vaccination data from vaccination history as recalled by the child’s mothers/caregivers.

#### Training of interviewers

Twenty-five interviewers were recruited. They were all students. They were given a two-day training course. The first was devoted to the presentation of the target diseases of the EPI, the vaccination schedule, HBR and the HFR. The second day was dedicated to the presentation of the survey objectives, the questionnaire administration method and the collection tools. A round table was made to read the questions. A question-and-answer game followed to further clarify the concepts. Next, the ODK collect application was presented. The teaching techniques used were PowerPoint presentation and role playing.

#### Collection method

Data collection began after verifying that the child belonged to the age group concerned by the survey. The interviewer determines the exact age of the child based on the date of birth given by the mother / caregiver and the date of the survey. The age is calculated automatically once the date of birth is entered into the tablet.

For each eligible child, two data sources are systematically used. The first is the HBR to collect vaccination status and date of vaccination. Investigators are encouraged to take pictures of HBR when they were unable to decipher vaccination dates.

The second source is the mother/caregiver statement to assess immunization status only. The questions were worded by describing the sites of administration of the vaccine, namely, the arm, shoulder, thigh and mouth in order to facilitate the understanding of mothers or caregivers.

Consultation of the HFR at the child’s usual health care facility is required in one of the following situations: i) the HBR is not available, ii) the HBR is available but information on vaccination is not there or not mentioned, is illegible or is incomplete. To facilitate the use of the HFR, the investigators recorded the first name, last name and date of birth of the child, as well as the first and last name of the mother before leaving the household. This information is sent, using WhatsApp, to the District Medical Officer (DMO) who, in turn, passes it on to the head of the health facility attended by the child. The latter, once the message has been received, checks the vaccination status and the date of vaccination. This data is transmitted to the DMO again and entered into the tablets.

#### Study variables

Vaccination data relate to vaccination status and date of vaccination. The dependent variable was HepB-BD vaccination coverage within 24 h. The delay was obtained by making the difference between the date of vaccination and the date of birth. It is binary and the modalities are: yes and no. Timely vaccination is one that has taken place within 24 h of birth. Because the time of birth is not marked on the vaccination documents, any vaccination that took place on the day of birth or the next day is considered to have occurred within 24 h after birth.

The explanatory variables are those identified and organized using Andersen's theoretical model:

Predisposing variables: Mother's age (< 20 years old, 20–29 years old and > 30 years old), mother's education (yes, no), marital status (married, unmarried), father's professional status (employed, unemployed), child’s sex (male, female), birth order (1–2 and > 2), knowledge about the age for beginning vaccination (yes, no), knowledge about the benefits of vaccination (yes, no), knowledge about co-administration (yes, no),

Enabling variables: place of residence (urban, rural), distance between residence and health facility (< 5 km, ≥ 5 km), access to newspaper (yes, no), access to radio (yes, no), access to television (yes, no), decision-making about the child's health (mother, husband/partner, mother and partner and others), family allowance (yes, no), health insurance (yes, no), wealth quintile (poorest, poor, middle, richer, richest), availability of the HBR (yes seen, yes but not seen, no), timely initiation of BF (≤ 1 h after birth, between 1 and 24 h after birth, beyond 24 h after birth), place of delivery (health facility, outside health facility), year of birth (2018, 2019), season of birth (dry season, rainy season).

Need variables: ANC attendance (< 4, ≥ 4), advice on vaccination during ANC (yes, no), Hospitalization of the newborn immediately after birth (yes, no), weighing of the newborn at birth (yes, no), gestational age (full term, prematurity), type of delivery (cesarean section, vaginal delivery), PNC attendance (yes, no, don't know), advice on vaccination during PNC (yes, no).

### Statistical analyzes

In accordance with the new WHO recommendations, the inverse probability weighting method was performed for the estimation of crude vaccination coverage with BCG, OPV-zero and HepB-BD. To this end, the probability of including a cluster was calculated. Then, in a given cluster, the probability of including a child is estimated. The product of these two probabilities was used to obtain the probability of selecting a child knowing that the cluster is selected. The reciprocal of this probability is the weight of each child included in the sample [31].

Two types of analyzes were performed. These are descriptive statistics and analytical statistics. The descriptive part made it possible to express the variables as a proportion.

Crude vaccination coverage is calculated for each of the three birth doses. This indicator is a proportion. The numerator is the weighted number of children who received the dose. The denominator is the weighted number of children who participated in the survey.

The vaccination status of the child is treated as follows: i) if the HBR is available with complete information, then it is valid, ii) if the HBR is not available but the child was in the HFR, then this is valid or iii) if the child does not have a HBR and his name does not appear in the HFR, mother / caregiver recall about the child’s vaccination history is valid.

Then, vaccination coverage within 24 h after birth is estimated as a proportion. For this indicator, only the HBR or the HFR are valid. The numerator is the number of children who received the vaccine within 24 h of life. The denominator corresponds to the number of children who received the vaccine regardless of the date of vaccination.

The analytical part consisted of investigating factors associated with timely HepB-BD vaccination. The choice made on this dose is explained by the fact that the administration of this dose within 24 h after birth is an indicator of EPI performance [23]. Bivariate analysis was performed using the Chi-square test with Rao and Scott’s correction. Then, the variables for which the p-value is less than or equal to 0.25 are integrated in a mixed effect multiple logistic regression model. Clusters were considered as random effect to account for the unexplained variability at the community level [33]. Variables are selected using the step-down procedure. The variance inflation factor (VIF) was examined to assess the collinearity between the explanatory variables. The significance level is set at 0.05. All the analyzes are carried out with the R software.

## Results

### Response rate

Of the 870 children aged 12 to 23 months planned, 832 were included, for a response rate of 95.86%.

### Basic characteristics of respondents

More than one in two mothers or caregivers were between 20 and 29 years old, at 54.7%. The proportion of married was 96.2%. That of children whose fathers were employed was 94.5%. Male children and those in the first or second birth order accounted for 53.0% and 45.2%, respectively.

In addition, the proportion of deliveries taking place in health facilities stands at 68.8%. In addition, 84.9% of mothers have attended PNC and 87.7% of children are weighed at birth (Table 1).

**Table 1:** Basic characteristics of respondents, Podor health district, June 2020 (n:unweighted *N* = 832, %:Weighted *N* = 8026)

Variables	*n*	%
Predisposing factors		
Maternal age		
< 20 years	110	12.6
20–29 years	439	54.7
> 30 years	283	32.6
Mother's education		
Yes	338	45.9
No	494	54.1
Mother’s marital status		
Married	805	96.2
Not married	27	3.8
Profession of the child's father		
Employment	790	94.5
Unemployed	42	5.5
Sex of child		
Male	430	53.0
Female	420	47.0
Birth order		
1–2	376	45.2
> 2	456	54.8
Knowledge about the age for beginning vaccination		
Yes	643	80.0
No	189	20.0
Knowledge about the benefits of vaccination		
Yes	749	92.6
No	83	7.4
Knowledge about co-administration		
Yes	571	72.1
No	261	27.9
Enabling factors		
Place of residence		
Urban	202	27.2
Rural	630	72.8
Distance		
< 5 km	534	70.1
≥ 5 km	298	29.9
Access to newspaper		
Yes	116	24.6
No	716	75.4
Access to radio		
Yes	582	73.8
No	250	26.2
Access to television		
Yes	360	46.9
No	472	53.1
Decision-making power		
Me/me and my husband	802	97.5
Other	30	2.5
Means of transport		
On walk	451	61.9
Transportations	381	38.1
Beneficiary of family allowance		
Yes	46	7.7
No	786	92.3
Access to health insurance		
Yes	41	5.0
No	791	95.0
Quintile		
Poorest	166	22.1
Poor	167	21.1
Middle	166	22.7
Richer	167	19.3
Richest	166	14.8
Availability of the HBR		
No	35	3.5
Yes not seen	56	5.3
Yes seen	741	91.2
Timely initiation of BF		
Immediately (≤ 1 h)	715	86.3
Beyond one hour	117	13.7
Place of birth		
Health facility	574	68.8
Home	258	31.2
Year of birth		
2018	419	48.7
2019	413	51.3
Season of birth		
Rainy season	187	20.3
Dry season	645	79.7
Need factors		
Number of ANC		
0–3	406	46.4
≥ 4	426	53.6
Advice on vaccination during ANC		
Yes	665	82.4
No	167	17.6
Hospitalization of the newborn		
Yes	193	23.6
No	639	76.4
Newborn weighing at birth		
Yes	695	87.7
No / don't know	137	12.3
Gestational age		
Full-term	816	98.0
Prematurity	16	2.0
Mode of delivery		
Cesarean section	82	12.9
Vaginal delivery	750	87.1
PNC		
Yes	677	84.9
No / don't know	155	15.1
Advice during PNC		
Yes	700	87.2
No / don't know	132	12.8

### Vaccination coverage

Two indicators were calculated. Crude BCG, HepB-BD and OPV-zero vaccination coverage were estimated at 95.2%, 88.1% and 89.3%, respectively. In contrast, vaccination coverage within the first 24 h of life was 13.9%, 42.1% and 30%, respectively (Table 2).

**Table 2:** Birth dose vaccination coverage among children aged 12 to 23 months, Podor health district (Unweighted *N* = 832, Weighted *N* = 8026)

Birth dose vaccines	VC according to HBR (a) %	VC according to HBR or HFR (b) %	VC according to history (c) %	Crude VC (b or c) %	VC within 24 h of life %
BCG	90.1	91.1	83, 4	95.2	13, 9
HepB-BD	82.3	84.1	74.4	88.1	42.1
OPV-zero	83.4	85	73.5	89.3	30

*VC* vaccination coverage, *HBR* home-based vaccination records, *HFR* Health facility register

Factors associated with HepB-BD vaccination within 24 h after birth.

### Bivariate analysis

In the bivariate analysis, the factors identified as associated with the administration of HepB-BD within 24 h after birth are: access to television, wealth quintile, institutional delivery, hospitalization of the child immediately after birth and weighing of the child at birth (Table 3). An additional file shows this in more detail [see Additional file 1].

**Table 3:** Bivariate analysis of factors associated with HepB-BD vaccination coverage within 24 h, (n:unweighted *N* = 629, %:Weighted *N* = 6417)

Variables	HepB-BD vaccination coverage within 24 h		*p* -value
	Yes *n* (%)	No *n* (%)	
Predisposing factors			
Child's mother's age (years)			0.894
< 20	38 (44.3%)	52 (55.7%)	
20–29	135 (40.7%)	191 (59.3%)	
> 30	92 (42.1%)	121 (57.9%)	
Mother’s education			0.503
No	137 (39.9%)	215 (60.1%)	
Yes	128 (43.5%)	149 (56.5%)	
Marital status			0.817
Unmarried	8 (38.0%)	15 (62.0%)	
Married	257 (41.7%)	349 (58.3%)	
Profession of the child's father			0.703
Employment	254 (41.8%)	344 (58.2%)	
Unemployed	11 (37.5%)	20 (62.5%)	
Sex of child			0.102
Female	118 (37.0%)	183 (63.0%)	
Male	147 (45.6%)	181 (54.4%)	
Birth order			0.474
> 2	140 (39.9%)	203 (60.1%)	
1—2	125 (43.7%)	161 (56.3%)	
Knowledge about the age for beginning vaccination			0.741
No	51 (39.9%)	72 (60.1%)	
Yes	214 (41.9%)	292 (58.1%)	
Knowledge about the benefits of vaccination			0.126
No	26 (53.4%)	27 (46.6%)	
Yes	239 (40.8%)	337 (59.2%)	
Knowledge about co-administration			0.869
No	70 (42.3%)	98 (57.7%)	
Yes	195 (41.4%)	266 (58.6%)	
Enabling factors			
Place of residence			0.834
Rural	185 (41.2%)	275 (58.8%)	
Urban	80 (42.5%)	89 (57.5%)	
Distance between home and health facility			0.214
≥ 5 km	83 (46.6%)	115 (53.4%)	
< 5 km	182 (39.9%)	249 (60.1%)	
Access to newspaper			0.044
No	224 (45.3%)	308 (54.7%)	
Yes	41 (31.9%)	56 (68.1%)	
Acces to Radio			0.014
No	70 (31.1%)	105 (68.9%)	
Yes	195 (45.1%)	259 (54.9%)	
Access to television			0.017
No	114 (35.4%)	220 (64.6%)	
Yes	151 (48.0%)	144 (52.0%)	

### Multivariate analysis

The multivariate analysis allowed to establish a statistically significant and independent effect of the variables named above, with the exception of the wealth quintile. Indeed, children whose mothers had access to television were 1.70 times more likely to receive HepB-BD on time (*p*-value = 0.012). In addition, this chance is multiplied by 1.62 (*p*-value = 0.046) and 3.90 (*p*-value < 0.001) when the child is born in a health facility and weighed at birth, respectively. In contrast, a newborn hospitalized immediately after birth has a 58% lower chance of receiving HepB-BD on time (*p*-value < 0.001) compared to an outpatient newborn (Table 4).

**Table 4:** Factors associated with HepB-BD vaccination coverage within 24 h, Podor health district, June 2020

Variables	OR _adjusted_	95% CI	*P*-value
Child’s sex			
Male	1.32	0, 92 – 1.91	0.133
Female	1		
Access to television			
Yes	1.70	1.12 – 2.57	0.012
No	1		
Place of birth			
Health facility	1.62	1.04 – 2.67	0.046
Home	1		
Hospitalization of newborn immediately after birth			
Yes	0.42	0.26 – 0.68	< 0.001
No	1		
Newborn weighing immediately at birth			
Yes	3.90	1.79 – 8.53	< 0.001
No / don't know	1		

## Discussion

This study showed that the crude vaccination coverage for BCG, OPV-zero dose and HepB-BD were 95.2%, 89.3% and 88.1%, respectively. On the other hand, vaccination coverage within 24 h of birth were estimated at 13.9%, 30% and 42.1%, respectively.

The factors associated with timely HepB-BD are delivery in a health facility, access to television, weighing and hospitalization of the newborn immediately after birth.

Crude vaccination coverage were higher than those observed nationally [25]. An Ethiopian study reported BCG and OPV-zero dose vaccination coverage equal to 72.9% and 18.9% according to the HBR and 77.7% and 25.1% according to the history [34]. A recent Mongolian study found, on the basis of data extracted from the vaccination card, crude vaccination coverage reaching 97.7%, 98.2% and 98.2% for BCG, OPV-zero dose and HepB-BD, respectively [35].

However, vaccination coverage within 24 h after birth is low with proportions estimated at 13.9%, 30% and 42.1% for BCG, OPV-zero dose and HepB-BD, respectively. Timely HepB-BD vaccination coverage is highest. Conversely, that of BCG is the weakest. Two explanations are possible. On the one hand, administration of HepB-BD within 24 h after birth is a performance indicator for EPI. On the other hand, the open vial policy varies from vaccine to vaccine. The HepB-BD vaccine is contained in a ten-dose vial which can be used for a maximum of four weeks after opening if and only if the storage conditions are met [36]. On the other hand, BCG is packaged in vials of twenty doses. The vial is only opened during sessions scheduled for at least 10 to 12 children due to the inability to use the doses beyond six hours after opening the vial. In the health district of Podor, BCG administration sessions are not done daily. Therefore, children born on a day when BCG services are unavailable are very unlikely to receive BCG vaccine within 24 h after birth. For OPV- zero dose, the schedule indicates that it can be given between the day of birth and day 14 of life. The absence of the "24 h" statement may contribute to the administration of the vaccine beyond this time.

In this study, many children are not weighed or have no proof of having been weighed at birth. Thus, the study is unable to determine the impact of birth weight on the timing of vaccination. Yet, in SSA, some health professionals have erroneously stated that they do not administer HepB-BD to low birth weight, sick or premature infants [15]. This study illustrate the importance of weighing births on timely immunization. According to the CDHS-2018, only 51.8% of children were weighed at birth in the northern part of the country [37]. This may explain the fact that the absence of weighing is a barrier to vaccination at birth.

Weighing and recording the weight on health documents (HBR or HFR) are part of immediate newborn care, while administration of HepB-BD is an essential newborn care [38]. Thus, an integration of the services responsible for providing these two types of care would make it possible to increase vaccination coverage at birth.

Hospitalization of the newborn immediately after birth has rightly appeared as a barrier to timely immunization. Two Vietnamese and Italian studies published in 2008 and 2014 respectively highlighted a similar situation [39, 40]. One explanation for this result from this current study could lie in the WHO recommendation that vaccination of newborns requiring resuscitation or other immediate care be delayed. Another explanation could be that health workers express reluctance to immunize unstable newborns, fearing that parents may falsely link adverse outcomes to the birth dose [38].

However, communication should be established between the originating and receiving institution to allow administration of the dose once the newborn is stable [38].

This study found that home birth is a barrier to immunization within 24 h of birth. Three studies from China, Ethiopia and Nigeria illustrate this phenomenon well [41–43], regardless of the socio-economic conditions of the mothers [42]. There is evidence that women who give birth in health facilities are those who live near them [41]. However, the health district of Podor is marked by its quasi-rural character and a large part of its population is located more than 5 km from health facilities [29]. The lack of mobile logistics can also hamper the holding of vaccination sessions in advanced or mobile strategy. This situation is the bedrock of the recurrence of home births, and consequently of the delay or absence of the administration of the vaccination from birth. In this regard, il would be to reach children born outside health facilities [44]. Success factors for immunizing children born at home are documented by WHO. These include the holding of home visits to provide the vaccine or other postnatal care, the monitoring of pregnancies at the community level by community health workers (CHWs) and the storage out-of-cold-chain (OCC) of vaccines but under controlled temperature chain (CTC) [44]. A study carried out in the Republic of Kiribati has shown that the identification, census and reporting of pregnant women or women in labor phase by the CHWs greatly improve vaccination coverage at birth among children born at home [45].

Additionally, the study found that access to television was positively associated with administration of HepB-BD within 24 h of birth. This result is evocative of the importance of the educational role of the media in public health. More and more people are interested in issues related to their health. Thus, the media are seen as a powerful channel for disseminating information and increasing vaccine awareness and the opinions of vaccine supporters and opponents [46]. This result is a reminder of the need for each country that has introduced HepB-BD in its EPI to put in place a general response plan against adverse events following immunization (AEFI), anti-vaccine movements, and any allegation likely to be harmful public acceptance of HepB-BD and confidence in the EPI [38]. This is all the more necessary as the rapid growth of the Internet and social networks have made it easier to research and disseminate concerns and misperceptions related to immunization [46]. For example, in Vietnam, it was observed a decrease in vaccination coverage with HepB-BD after media reports of AEFI that occurred following HepB-BD administration [47].

### Strengths and weaknesses

The strengths of this study are important to underline. First, the sample size and number of clusters were calculated according to the new WHO recommendations for immunization coverage surveys [31].

Cross-sectional studies are subject to selection and information bias, as well as to confounding factors [48]. In this present study, the circumstance likely to introduce selection bias is related to non-response. However, the use of weighting made it possible to control the bias. This is demonstrated in the literature [49, 50].

Then, the introduction of information bias is mitigated by three strategies. The first is the use of HFR when HBR is unavailable or does not provide accurate immunization status information. The second strategy is to choose a cohort of children aged 12 to 23 months, thus allowing mothers or caregivers to remember the vaccines received. Finally, the training of interviewers and the pre-test of the questionnaire made it possible to harmonize the data collection procedures. Kesmodel US considers that this method is one of the rare solutions to control this type of bias [51].

Confounding factors are a major source of bias [52]. In this study, socio-economic factors may emerge as confounding factors in the link between delivery in a health facility and timely vaccination. The effect of such confusion was controlled through the use of logistic regression. This demonstrated the stability of the link between childbirth in a health facility and the administration of the birth dose within 24 h of birth; whatever the socio-economic level.

This study has three main limitations. The first is due to its cross sectional nature, which does not allow a causal relationship to be established between the independent variables and the dependent variable. The second limitation relates to the geographic nature of the study. This was conducted in a single district which may have different characteristics compared to other health districts in the country. Therefore, the generalizability of the results should be viewed with caution. The third limitation is that the time of birth is not mentioned in the HFR. In this case, it is difficult to know precisely whether the child is vaccinated or not within 24 h of birth. The resulting consequence would be the overestimation of immunization coverage within 24 h of birth.

## Conclusion

This study indicated satisfactory crude vaccination coverage. But those for the 24-h postnatal period are very low. Factors favorable to timely vaccination were access to television, delivery in a health facility and weighing the newborn immediately after birth. In contrast, hospitalization of the newborn immediately after birth was found to be a barrier. Therefore, actions should focus on sensitizing mothers on the importance of immunization at birth using mass media such as television, conducting home visits and pregnancy monitoring by CHWs to administer timely birth doses to home-born children, integrating immunization at birth with essential neonatal care such as weighing, and creating linkages between neonatal immunization services and hospitalization services.

In addition, further studies should be conducted to increase knowledge about the facets of birth vaccination.

## Supplementary Information

**Additional file 1: Table 3.** Bivariate analysis of factors associated with HepB-BD vaccination coverage within 24 hours (N = 629), continued. **Table 3.** Bivariate analysis of factors associated with HepB-BD vaccination coverage within 24 hours (N = 629), continued and end.

## Data Availability

The data used to conduct this study is available from the corresponding author.

## Abbreviations

AEFI: Adverse event following immunization
ANC: Antenatal care
AOR: Adjusted odds ratio
BCG: Bacille Calmette et Guérin
BF: Breastfeeding
CDHS: Continuous Demographic and Health Survey
CHW: Community health workers
CTC: Controlled temperature chain
EPI: Expanded programme on imminuzation
HBR: Home-based record
HBV: Hepatitis B virus
Hep-BD: Birth dose of hepatitis B vaccine
HFR: Health facility registry
OCC: Out-of-cold-chain
OPV-zero: Birth dose of oral polio vaccine
PNC: Postnatal care
SSA: Sub-Saharan Africa
VIF: Variance inflation factor
WHO: World Health Organization
