# Supplementary material for: Vaccination against tuberculosis, polio and hepatitis B at birth in Podor health district, Northern Senegal: cross-sectional study of vaccination coverage and its associated factors
Source: BMC Public Health. 2022 Jan 15;22:110. doi: 10.1186/s12889-022-12535-z (PMC8761353; doi:10.1186/s12889-022-12535-z)
Supplement: Supplementary file 1 — Additional file 1: Table 3. Bivariate analysis of factors associated with HepB-BD vaccination coverage within 24 hours (N = 629), continued. Table 3. Bivariate analysis of factors associated with HepB-BD vaccination coverage within 24 hours (N = 629), continued and end. [file 12889_2022_12535_MOESM1_ESM.docx]

Table 3 : Bivariate analysis of factors associated with HepB-BD vaccination coverage within 24 hours (N = 629), continued

| Variables | **HepB-BD vaccination coverage within 24 hours** | | **p -value** |
| --- | --- | --- | --- |
|  | **Yes** | **No** |  |
| Decision-making power |  |  | 1,000 |
| Other | 8 (40.0%) | 12 (60.0%) |  |
| Me / Me and my husband | 257 (42.2%) | 352 (57.8%) |  |
| Means of transport |  |  | 0.170 |
| On walk | 166 (44.5%) | 207 (55.5%) |  |
| Transportations | 99 (38.7%) | 157 (61.3%) |  |
| Family allowance |  |  | 0.318 |
| No | 246 (41.6%) | 346 (58.4%) |  |
| Yes | 19 (51.4%) | 18 (48.6%) |  |
| Health coverage |  |  | 0.098 |
| No | 247 (41.3%) | 351 (58.7%) |  |
| Yes | 18 (58.1%) | 13 (41.9%) |  |
| Quintile |  |  | 0.006 |
| Poor | 136 (48.6%) | 144 (51.4%) |  |
| Middle | 53 (41.4%) | 75 (58.6%) |  |
| Rich | 76 (34.4%) | 145 (65.6%) |  |
| Availability of the HBR |  |  | 0.315 |
| No | 3 (75.0%) | 1 (25.0%) |  |
| Yes | 262 (41.9%) | 363 (58.1%) |  |
| Timely initiation of BF |  |  | 0.409 |
| Beyond one hour | 42 (46.7%) | 48 (53.3%) |  |
| Immediately (≤1hour) | 223 (41.4%) | 316 (58.6%) |  |
| Place of birth |  |  | <0.001 |
| Home | 42 (27.3%) | 112 (72.7%) |  |
| Health facility | 223 (46.9%) | 252 (53.1%) |  |
| Year of birth |  |  | 0.610 |
| 2018 | 124 (40.9%) | 179 (59.1%) |  |
| 2019 | 141 (43.3%) | 185 (56.7%) |  |
| Season of birth |  |  | 0.926 |
| Dry season | 208 (41.9%) | 288 (58.1%) |  |
| Rainy season | 57 (42.9%) | 76 (57.1%) |  |
| **Needs factors** |  |  |  |
| Number of ANC |  |  | 0.079 |
| <4 | 110 (38.2%) | 178 (61.8%) |  |
| ≥ 4 | 155 (45.5%) | 186 (54.5%) |  |
| Advice on vaccination during ANC |  |  | 1,000 |
| No / Do not know | 46 (42.2%) | 63 (57.8%) |  |
| Yes | 219 (42.1%) | 301 (57.9%) |  |
| Hospitalization of the newborn immediately after birth |  |  | <0.001 |
| No | 222 (47.1%) | 249 (52.9%) |  |
| Yes | 43 (27.2%) | 115 (72.8%) |  |
| Newborn weighing at birth |  |  | <0.001 |
| No / Do not know | 10 (15.6%) | 54 (84.4%) |  |
| Yes | 255 (45.1%) | 310 (54.9 |  |

Table 3 : Bivariate analysis of factors associated with HepB-BD vaccination coverage within 24 hours (N = 629), continued and end

| Variables | **HepB-BD vaccination coverage within 24 hours** | | **p -value** |
| --- | --- | --- | --- |
|  | **Yes** | **No** |  |
| Gestational age |  |  | 0.742 |
| Full-term | 258 (42.0%) | 357 (58.0%) |  |
| Prematurity | 7 (50.0%) | 7 (50.0%) |  |
| Mode of delivery |  |  | 0.794 |
| vaginal delivery | 231 (41.8%) | 321 (58.2%) |  |
| Cesarean section | 34 (44.2%) | 43 (55.8%) |  |
| PNC |  |  | 0.069 |
| No / Do not know | 28 (32.6%) | 58 (67.4%) |  |
| Yes | 237 (43.6%) | 306 (56.4%) |  |
| Advice on vaccination during PNC |  |  | 0.593 |
| No / Do not know | 31 (38.8%) | 49 (61.3%) |  |
| Yes | 234 (42.6%) | 315 (57.4%) |  |
